# Supplementary material for: A novel mechanism of “metal gel-shift” by histidine-rich Ni2+-binding Hpn protein from Helicobacter pylori strain SS1
Source: PLoS One. 2017 Feb 16;12(2):e0172182. doi: 10.1371/journal.pone.0172182 (PMC5312948; doi:10.1371/journal.pone.0172182)
Supplement: S1 File — (PDF) [file pone.0172182.s001.pdf]

S1 Supporting Information for Manuscript- Shelake *et al*

A novel mechanism of "metal gel-shift" by histidine-rich Ni^{2+}-binding Hpn protein from *Helicobacter pylori* strain SS1

Rahul Mahadev Shelake[1], Yuki Ito[1], Junya Masumoto[1], Eugene Hayato Morita[2,3], Hidenori Hayashi[1]*

[1]Proteo-Science Center, Ehime University, Matsuyama, 7908577, Japan

[2]Laboratory of Molecular Cell Physiology, Faculty of Agriculture, Ehime University, Matsuyama, 7908566, Japan

[3]Department of Chemistry, Faculty of Science, Josai University, Saitama, 3500295, Japan

***Corresponding Author:**

E-mail: hayashi.hidenori.mj@ehime-u.ac.jp (HH)

Material and Methods section

A. *H. pylori* strain and growth conditions

The mouse-adapted strain of *Helicobacter pylori* Sydney strain (*H. pylori* SS1) was used in this study (Lee *et al.* 1997). *H. pylori* SS1 was grown on Tripticase Soy agar with 5% sheep blood (TSA II) (Becton Dickinson, Franklin Lakes, NJ) for three days at 35ºC in 12% carbon dioxide condition. A single colony was isolated and sub-cultured on TSA II agar at 35ºC in 12% carbon dioxide condition.

B. Purification of DNA from *H. pylori* strain SS1

The genomic DNA from strain SS1 was extracted based on phenol-chloroform method as described previously (Lee *et al.* 1997) with minor modifications. Briefly, bacteria were harvested from TSA II agar and suspended in 3 ml of Tris-buffered saline (TBS). After washing with TBS once by centrifugation at 3,800 g for 5 min at 4ºC, 5 x 10^8 colony forming unit (CFU) of bacteria were re-suspended in 500 µl of lysis buffer (50 mM Tris-HCl, pH 8.0; 100 mM EDTA, pH 8.0; 1 % SDS, 100 mM NaCl) containing 0.2 mg/ml proteinase K (Amaresco, Solon, OH) and incubated at 37ºC for 12 h. Subsequently, UltraPure™ buffer-saturated phenol (Invitrogen, Carlsbad, CA) was added, and the mixture was gently rotated for 15 min. After centrifugation, the aqueous phase was transferred to a tube containing chloroform/isoamyl alcohol (24:1, Sigma-Aldrich, St. Louis, MO) and agitated gently for 10 min. The aqueous phase was collected to a new tube by centrifugation. The DNA was precipitated by addition of isopropyl alcohol and 0.3 M sodium acetate, and then the DNA pellet was rinsed with 70% ethanol. After centrifugation, pellet was air-dried and dissolved in Tris-EDTA buffer (pH 8.0).

C. Enzyme-linked immunosorbent assay (ELISA) analysis

Green fluorescent protein with artificial His.tag (*gfp-His*$_6$) and GFP fused with Hpn (*gfp-*

Hpn) were cloned in pET21b. IPTG-induced over-expression of both the proteins was done with or without Ni^{2+} added in the culture (**Fig S3-A and B**). Pellets of 60 µl bacterial cultures were dissolved in 60 µl sample buffer and incubated for 3 min at 100ºC. Final volume of 15 µl loaded in each lane for SDS-PAGE. ELISA experiment was performed using previously described protocol with some modifications (Miura *et al.* 2008). Different buffers were prepared before starting ELISA experiment (**buffer components summarized in Table D**).

Flat-bottom 96-well ELISA plates (untreated 96-well microplates from Falcon) were used for coating. Concentration of each protein (Hpn, GFP-Hpn and GFP-His_6) was adjusted to 1 µg by dilution with coating buffer to the final volume of 50 µl. Plates were incubated at 4ºC for overnight. Next day, solution was thrown away and 200 µl blocking buffer into each well was added. Then, plate was incubated at 37ºC for 1hr. After incubation, solution was discarded and plate was washed three times by washing buffer. His.Tag® antibody was diluted to standardized concentration (1:500) with dilution buffer [C-terminal specific-anti 6xhistidine monoclonal antibody (9F2) (Wako Japan, product code: 010-21861)] and plate incubated at 37ºC for 1hr after adding 100 µl in each well. After three washes with wash buffer, plate was incubated with horseradish peroxidase-conjugated anti-mouse IgG (GE Healthcare, product code: NA931VS, diluted to 1:1000 in wash buffer) for 1 hr at 37ºC. After similar washing, the substrate ABTS [2,2'-Azinobis(3-ethylbenzothiazoline-6-sulfonic Acid Ammonium Salt) from Wako Japan] dissolved in 0.1 M citrate buffer and hydrogen peroxide (0.03%) was added. Then the plate was incubated for 20 minutes at room temperature. Reactions were stopped by adding stop buffer (100 µl) in each well. The absorbance at 415 nm was measured using a Spectramax M3 microplate reader (Molecular Devices Co., Sunnyvale, CA). Values obtained (absorbance at 415 nm) for Ni^{2+}-treated samples (from average of at least three replications) were normalized against untreated samples and plotted in graph.

Result section

Metal-binding to Hpn changes Hpn-antibody interaction

Interaction of His.Tag® antibody with Hpn may not necessarily show similar results for other protein having artificial His.Tag or Hpn conjugated with another protein owing to differential co-ordination geometry of metal-binding and its chemical surrounding. We investigated this possibility using GFP- His_6 and GFP-Hpn (**Fig S3-A and B**). GFP does not interact with His.Tag® antibody by its own. GFP is comparatively large protein (26.7 kDa) but still positional shift was observed in case of GFP-Hpn expressed in LB medium supplied with Ni^{2+}. Western blot data of GFP-His_6 and GFP-Hpn showed almost equal intensity signals in both the cases i.e. with or without Ni^{2+} (**Fig S3-C**).

Recognition site for His.Tag® antibody is His_6 peptide attached at C-terminal of a recombinant protein and it is a linear epitope. Hence, conformational change upon Ni^{2+}-binding to Hpn may lead to altered binding of His.Tag® antibody. This was further examined by ELISA using His.Tag® antibody (**Fig S3-D**). The SDS-treated and non-treated Hpn protein shown similar results which is compatible with previous observations for several other proteins (Lechtzier *et al,* 2002; Burgass *et al,* 2008). The relative detection sensitivity of Ni^{2+}-treated protein with His.Tag® antibody in ELISA give order of untagged Hpn < GFP-Hpn < GFP-His_6. Hence, the variability that we observed in detection of apo- and metalated-Hpn on western blots may have resulted not only from membrane-binding efficiency but also from differential exposure of His-rich region upon metal-binding.

These data signify that the metal-binding to Hpn causes altered binding of His.Tag® antibody, possibly due to change in protein confirmation.

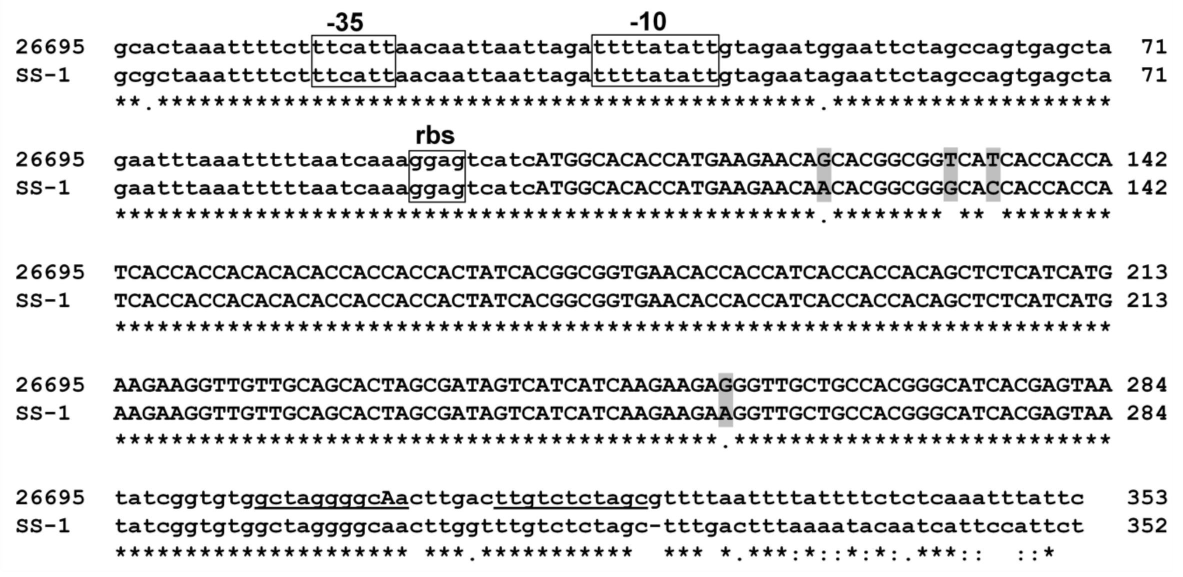

Fig A. Comparison of DNA sequence of *hpn* from *Helicobacter pylori* strain SS1 (this study) with strain 26695 (NCBI data).

The promoter region and *hpn* gene of strain SS1 was PCR amplified and nucleotide sequence was compared with NCBI data of strain 26695 (GenBank accession number U26361). Putative promoter elements are shown in a box. The *hpn* gene region is shown in uppercase letters and mutations at the nucleotide level are shaded in gray. The putative terminator region of transcription is underlined. The promoter region was highly conserved in strain SS1 including Shine-Dalgarno sequence (GGAG) and promoter elements (-10 and -35).

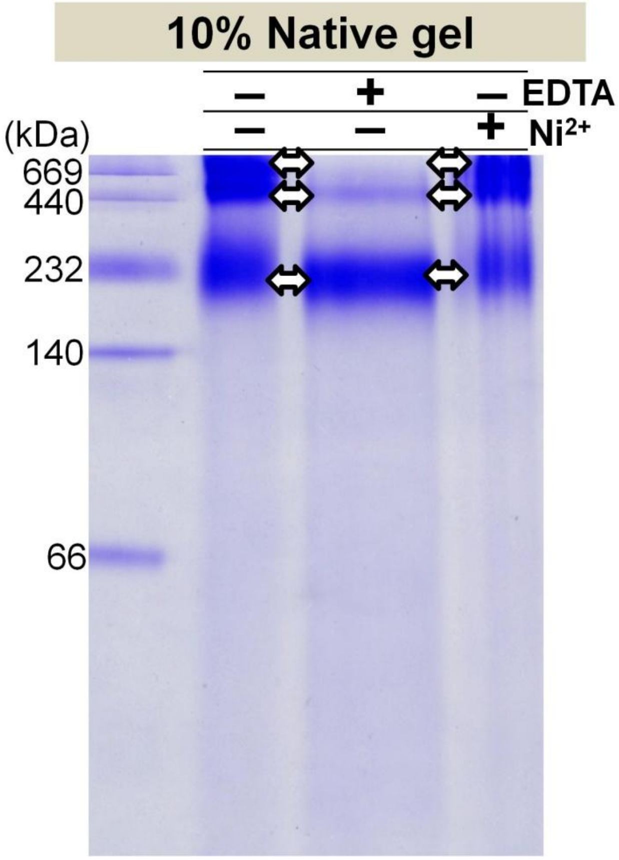

Fig B. Separation of purified Hpn on non-denaturing blue native-PAGE.

High molecular weight marker (GE Healthcare), abbreviated as HMW, was used in all gels [Thyroglobulin (669 kDa), Ferritin (440 kDa), Catalase (232 kDa), Lactate dehydrogenase: (140 kDa) and Albumin (66 kDa)].

Recombinant Hpn (200 µM) was treated with either 1 mM of EDTA or Ni^{2+} independently and then applied to 10% native-gel. Apparent multimeric complexes of >670, ~500, ~230 kDa were observed in presence or absence of Ni^{2+} and EDTA.

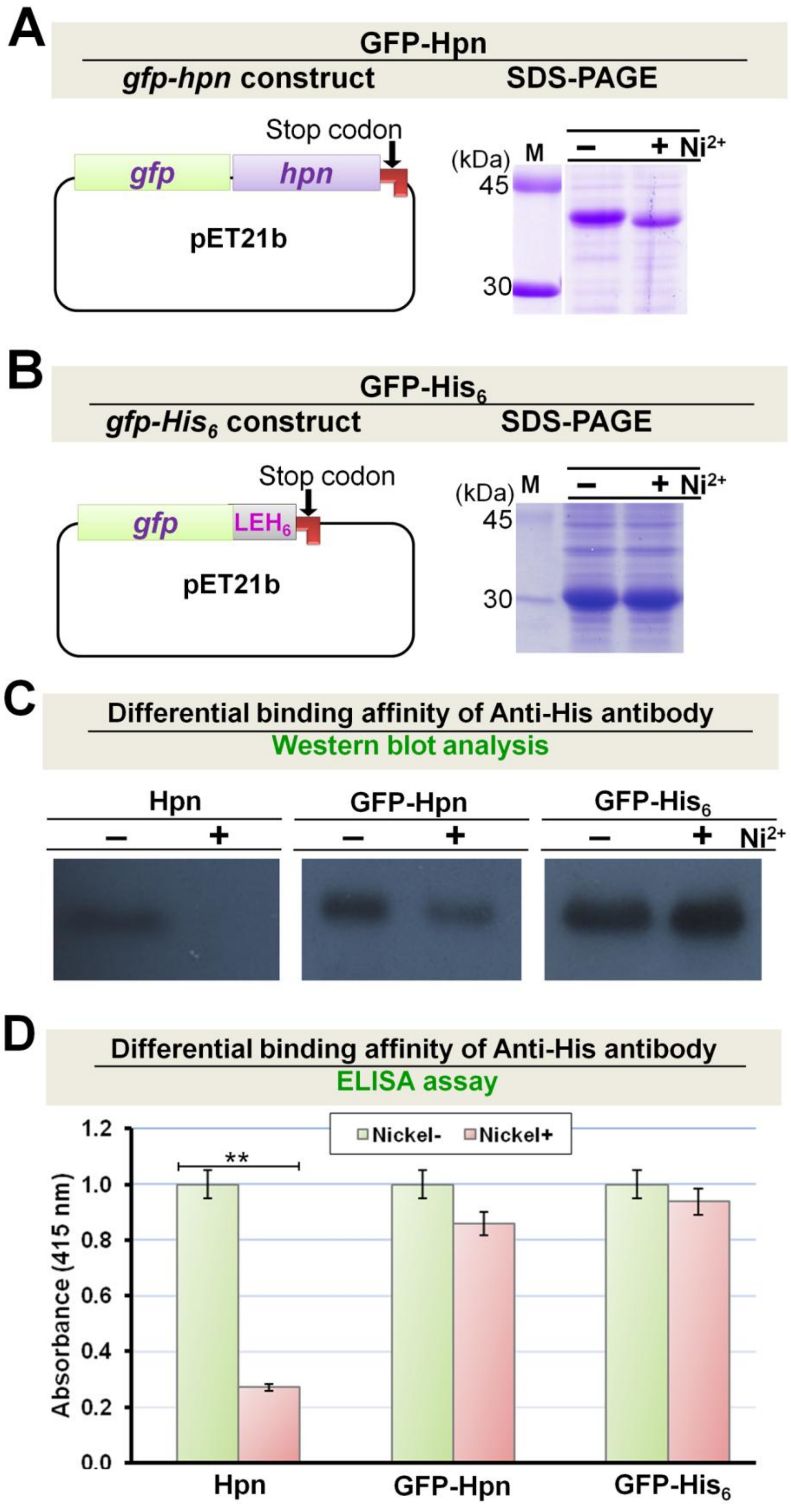

Fig C. Western blot and ELISA analysis of Hpn, GFP-His$_6$ and GFP-Hpn.

A. Schematic diagram of gene construct consisted of *gfp* fused with artificial His.tag (left panel) and GFP-His6 protein expression confirmed by SDS-PAGE (right panel).

B. Schematic diagram of *gfp* fused with *hpn* at C-terminal followed by stop codon (left panel) and recombinant protein of GFP-Hpn fusion analyzed by SDS-PAGE (right panel).

C. Western blot of untagged Hpn, GFP-Hpn and GFP-His_6 on PVDF membrane either treated with or without Ni^{2+} solution (1mM of $NiSO_4$).

D. ELISA of denatured untagged Hpn, GFP-Hpn and GFP-His_6 Equal amount of protein (1 μg) was coated on ELISA plate. His.Tag® antibody was diluted to 1:500 for detection. Values obtained (absorbance at 415 nm) for Ni^{2+}-treated samples (from average of at least three replications) were normalized against untreated samples and plotted in graph. Paired t-test was performed to compare the metal ion effect, ** indicate $p<0.01$.

Table A.

Proteins showing apparent SDS-resistant oligomeric forms upon denaturing SDS-PAGE

Protein	Oligomeric form	Remark	Reference
Na^+/H^+ exchanger protein 1	High-molecular-weight aggregate	Aggregated when subjected to elevated temperature	Bullis *et al.* 2002
Amyloid β (Aβ) protein	Dimer, trimer, tetramer	Copper induced oligomerization on SDS-PAGE	Atwood *et al.* 2000; 2004
Inducible nitric oxide synthase (iNOS)	Dimer	Human iNOS expressed *in vivo* only form undisruptable dimer	Kolodziejski *et al.* 2003
Acid phosphatase	Tetramer	Retained in partially denatured conditions	Tham *et al.* 2010
Bovine α-lactalbumin (α-LA), hen egg lysozyme (LYS) and human serum albumin (HSA)	Several forms depending on Pt concentration	Oligomerization of proteins was promoted by Pt-protein adduct formation	Pinato *et al.* 2013
Superoxide dismutase	Hetermeric form of ~125 kDa	Retained a small fraction of a multimeric form along with monomer	Nowakowski *et al.* 2014
Hpn	High-molecular-aggregate	Observed in -elution buffer containing higher imidazole	Present study

Table B.

List of proteins retaining metal ion on SDS-PAGE.

**Protein**	**Detection method**	**Metal ion retained**	**Remark**	**Reference**
**A. Denaturing SDS-PAGE**				
Sub-unit of proteins from a photochemical center	SDS-PAGE, Particle induced X-ray emission	Fe	Direct detection in the gel bands.	Solis *et al.* 1998
Ca^{2+}-dependent protein kinase from Soybean	SDS-PAGE	Ca	Ca^{2+}-dependent shift of electrophoretic mobility	Li *et al.* 1998
Ca^{2+}-dependent protein kinase from Tobacco	SDS-PAGE	Ca	Ca^{2+}-dependent shift of electrophoretic mobility	Yoon *et al.* 1999
AtCaM8 and AtCaM9 from Arabidopsis	SDS-PAGE	Ca	Ca^{2+}-dependent electrophoretic mobility shifts	Köhler and Neuhaus, 2000
Cisplatin-treated *E. coli* cells	LA-ICP-MS, RP-ESI-Q-TOF	Pt	Identification of Pt-containing proteins	Allardyce *et al.* 2001
Proteins from human liver cytosol	Synchrotron radiation X-ray fluorescence	Zn, Cu, Fe	Gel was dried immediately after electrophoresis	Gao *et al.* 2003
Cyanobacterial SmtA	LA-ICP-QMS	S, Zn, Cd	Measured directly from bacterial cell extracts resolved on SDS-gel	Binet *et al.* 2003
Apoazurin	Synchrotron radiation X-ray fluorescence	Zn	Zinc detected in CBB-stained protein band.	Weseloh *et al.* 2004
Proteins from *in vitro* callus of Citrus	SDS-PAGE with Synchrotron radiation X-ray fluorescence	Ca, Cu, Fe, K, Mg, Na and Zn	Microwave oven used for protein bands decomposition	Verbi *et al.* 2005
Albumin-depleted bovine serum proteins.	synchrotron X-ray fluorescence mapping with micro-XANES	Cr	Chromium-protein interactions allowed better seperation	Finney *et al.* 2010
Ca^{2+}-dependent protein kinase from Arabidopsis	SDS-PAGE	Ca	Ca^{2+}-dependent shift of electrophoretic mobility	Romeis *et al.* 2011
Bovine α-lactalbumin (α-LA), hen egg lysozyme (LYS) and human serum albumin (HSA)	SDS-PAGE	Pt	SDS-resistant protein oligomers	Pinato *et al.* 2013
Superoxide dismutase	LA-ICP-MS	Cu	Minor fraction retained	Nowakowski *et al.* 2014
Ferredoxin II in *Mesorhizobium loti*	SDS-PAGE	Ca	Ca^{2+}-dependent shift of electrophoretic mobility	Moscatiello *et al.*2015
Hpn	SDS-PAGE, MALDI-TOF-MS, ICP-OES	Ni	Ni^{2+}-dependent shift of electrophoretic mobility	Present study
**B. Modified SDS-PAGE conditions**				
Human brain proteins	MALDI-FT-ICR-MS and LA-ICPMS	Zn, Cu	Second dimension IEF (SDS-PAGE)	Becker *et al.* 2005
Yeast mitochondrial ATPase	LA-ICP-MS and MALDI-FT-ICR-MS	Fe, Zn, Cu	Second dimension IEF (SDS-PAGE)	Krause-Buchholz *et al.* 2006
Superoxide dismutase, alcohol dehydrogenase	SDS-Tris-Tricine-PAGE-LA-ICP-MS	Cu, Zn	Lower current for separation and without CBB-staining	Jiménez *et al.* 2010
Human serum albumin (HSA)	OFFGEL isoelectric focusing and ICP-MS	Pt	SDS-PAGE in the absence of BME or DTT	Mena *et al.* 2011
Protein fractions of a rat kidney cytosol treated with	nLC–ESI-LTQ-MS/MS	Pt	TCEP (Tris 2-carboxyethyl	Mena *et al.* 2013

oxaliplatin			phosphine)-based SDS-PAGE	
SOD,Yeast ADH, Bovine AP, Carbonic anhydrase, β-galactosidase	LA-ICP-MS, in-gel Zn-protein staining	Cu, Zn	Lower SDS in buffer (0.0375%) plus no EDTA in buffer	Nowakowski *et al*. 2014

Table C.

List of non-covalent protein-metal ion interactions studied by MALDI in combination with other methods.

Peptide	Metal	Matrix solution	Combined method	Reference
$(GHHPH)_5G$ peptide	Cu	DHB	TOF-MS	Hutchens *et al.* 1991
Human glycoprotein	Cu	DHB in 0.1% aqueous TFA	TOF-MS	Nelson and Hutchens 1992
Zinc finger peptides	Zn	HCCA in 1:l ammonium bicarbonate (1M)-ethanol.	-	Woods *et al.* 1995
Prion protein	Cu	DHB and 6,7-dihydroxycoumarin	TOF-MS	Hornshaw *et al.* 1995
Ferrichrome	Fe	DHB in methanol	TOF-MS	Kaltashov *et al.* 1997
Luteinizing hormone releasing hormone	Ni, Cu, Zn	Paranitroaniline in ethanol (10 mg/mL)	Fourier Transform-MS	Masselon *et al.* 1999
Zinc finger peptide	Zn	6-aza-2-thiothymine or DHB in Tris (10 mM), ammonium bicarbonate (20 mM) or 0.1% TFA	TOF-MS	Lehmann *et al.* 1999
Bradykinin	Cu, Ag, Co, Ni, Zn	HCCA matrix saturated in water and acetonitrile (70:30 v/v) containing 0.1% TFA.	TOF-MS	Cerda *et al.* 1999
Prion proteins	Cu	Sinapinic acid in 20% acetonitrile	TOF-MS	Qin *et al.* 2002
Human brain proteins	Cu, Zn	DHB in acetonitrile and 0.1% TFA in water (2:1)	FT-ICR -MS	Becker *et al.* 2005
Human tau proteins	Cu, Zn	DHB in acetonitrile and 0.1% TFA in water (2:1)	FT-ICR -MS	Susanne Becker *et al.* 2007
Human brain proteins	P, Cu, Zn, Fe	DHB in acetonitrile: 0.1% TFA in water (2:1)	FT-ICR-MS	Becker *et al.* 2007
Angiotensin I	Cu, K	HCCA, 3-aminoquinoline and glycerol	TOF-MS	Hortal *et al.* 2008
Rat tissues	Zn, Cu, Fe, Cr, Cd, Pb	HCCA in acetonitrile:0.1% TFA in water (2:1)	TOF-MS and LA-ICP-MS	Becker *et al.* 2008

a-Crystallin	Zn	HCCA in 1:2 acetonitrile and 0.1 % TFA	TOF-MS	Karmakar and Das 2012
Protein fraction from Brazil nuts	Cu	HCCA in 50% acetonitrile and 0.1% TFA	TOF-MS	Jayasinghe and Caruso 2013
Hpn	Ni	Sinapinic acid (100% ACN, 0.01% TFA, and distilled water; v:v, 50:10:40)	TOF-MS	Present study

(DHB: 2,5-dihydroxybenzoic acid; HCCA: α-cyano-4-hydroxycinnamic acid; FT-ICR-MS: Fourier transform-ion cyclotron resonance-mass spectrometry; LA-ICP-MS: Laser ablation-inductively coupled plasma-mass spectrometry)

Table D.
Components of different buffers used in ELISA.

Buffer type	Components
Coating buffer	Phosphate buffer saline, [1.16 g Na_2HPO_4, 0.1g KCl, 0.1 g K_3PO_4, 4.0 g NaCl (500 ml distilled water) pH 7.4]
Dilution buffer	0.1% BSA with 0.05% Tween-20 in PBS
Blocking buffer	1% BSA with 0.05% Tween-20 in PBS
Washing buffer	0.05% Tween-20 in PBS
Stop buffer	5N Sodium Hydroxide in distilled water

Annexure A.

Protocol followed for analysis of apparent MW of Hpn on SDS-PAGE in Fig. 5

(Important commands are mentioned in bracket)

1. Picture file opened in ImageJ software (File – open or drag to icon).
2. Brightness/contrast was adjusted for easy comparison (Image - Adjust-) and picture was Inverted (Edit - Invert).

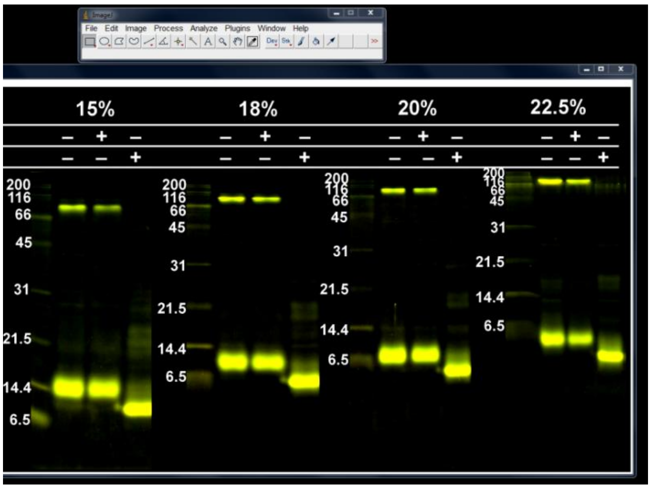

3. Shape for selection was chosen as straight line (width 30) by double click on the icon.
4. Measurement parameters were selected (Analyze – Set Measurements – Select parameters – click add to overlay and length).
5. Measurements were done by selecting area from top of the gel till the specific protein separated on gel (Analyze – Measure).

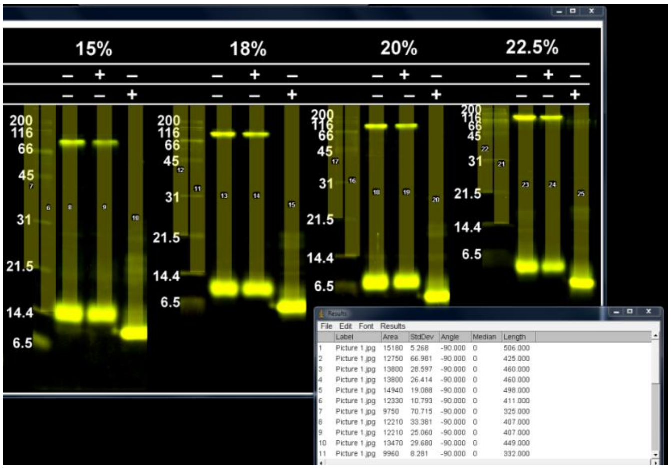

6. All values saved to excel file.

7. Apparent MW on SDS-gel was calculated on the basis of known theoretical MW for two marker proteins- lysozyme (14.4 kDa) and trypsin inhibitor (21.5 kDa). Formulae used as follows-

A. Difference in migration distance (DMD) compare to (14.4 kDa) marker protein = MD from top of gel by Lysozyme (14.4 kDa) – MD from top of gel of respective Hpn band.

B. MW difference compare to 14.4 kDa band

$$= \frac{\text{(DMD compare to 14.4 kDa for Hpn band) x 7.1}}{\text{(DMD compare to 14.4 kDa for 21.5 kDa band)}}$$

Factor 7.1 is the MW difference between two marker proteins i.e. lysozyme and trypsin inhibitor (21.5–14.4=7.1)

C. Apparent MW (kDa) = 14.4 kDa + MW Difference for (EDTA or Ni) Hpn (kDa)

For example, for apparent MW for Ni^{2+}-Hpn on 12.5% gel = 14.4 + 0.70 = 15.1 kDa

Gel %	Protein band on SDS-gel	Distance from top of gel	DMD compare to 14.4 kDa band	MW difference compare to 14.4 kDa band	Apparent MW (kDa)
**15.0%**	14.4 kDa	411			14.40
	21.5 kDa	325	86	7.10	21.50
	Apo-Hpn	407	4	0.33	14.73
	EDTA-Hpn	407	4	0.33	14.73
	Ni^{2+}-Hpn	449	-38	-3.14	11.26
**18.0%**	14.4 kDa	332			14.40
	21.5 kDa	259	73	7.10	21.50
	Apo-Hpn	360	-28	-2.72	11.68
	EDTA-Hpn	360	-28	-2.72	11.68
	Ni^{2+}-Hpn	396	-64	-6.22	8.18
**20.0%**	14.4 kDa	300			14.40
	21.5 kDa	224	76	7.10	21.50
	Apo-Hpn	350	-50	-4.67	9.73
	EDTA-Hpn	350	-50	-4.67	9.73
	Ni^{2+}-Hpn	386	-86	-8.03	6.37
**22.5%**	14.4 kDa	235			14.40
	21.5 kDa	174	61	7.10	21.50
	Apo-Hpn	317	-82	-9.54	4.86
	EDTA-Hpn	317	-82	-9.54	4.86
	Ni^{2+}-Hpn	352	-117	-13.62	0.78

References

References for Material and Methods section

[1] Lee A, Rourke JO, Ungria MCDE, Robertson B, Daskalopoulos G, Dixon MF. A standardized mouse model of Helicobacter pylori infection: introducing the Sydney strain. 1997; 112: 1386–1397. PMID: 9098027

[2] Miura K, Orcutt AC, Muratova O V., Miller LH, Saul A, Long CA. Development and characterization of a standardized ELISA including a reference serum on each plate to detect antibodies induced by experimental malaria vaccines. Vaccine. 2008; 26: 193–200. doi:10.1016/j.vaccine.2007.10.064

References for result section

[3] Lechtzier V, Hutoran M, Levy T, Kotler M, Brenner T, Steinitz M. Sodium dodecyl sulphate-treated proteins as ligands in ELISA. J Immunol Methods. 2002; 270: 19–26. doi:10.1016/S0022-1759(02)00214-4

[4] Burgess NK, Dao TP, Stanley AM, Fleming KG. β-Barrel Proteins That Reside in the Escherichia coli Outer Membrane in Vivo Demonstrate Varied Folding Behavior in Vitro. J Biol Chem. 2008; 283: 26748–26758. doi:10.1074/jbc.M802754200

Reference in Supplementary Table A

[5] Bullis BL, Li X, Rieder CV, Singh DN, Berthiaume LG, Fliegel L. Properties of the Na+/H+ exchanger protein. Eur. J. Biochem. 2002; 269: 4887-4895. http://dx.doi.org/10.1046/j.1432-1033.2002.03202.x

[6] Atwood CS, Scarpa RC, Huang X, Moir RD, Jones WD, Fairlie DP, Tanzi RE, Bush AI. Characterization of copper interactions with Alzheimer amyloid β peptides. J. Neurochem. 2000; 75: 1219–1233. http://dx.doi.org/10.1046/j.1471-4159.2000.0751219.x

[7] Atwood CS, Perry G, Zeng H, Kato Y, Jones WD, Ling KQ, Huang X, Moir RD, Wang D, Sayre LM, Smith MA. Copper mediates dityrosine cross-linking of Alzheimer's amyloid-β. Biochemistry. 2004; 43: 560–568. http://dx.doi.org/10.1021/bi0358824

[8] Kolodziejski PJ, Rashid MB, Eissa NT. Intracellular formation of" undisruptable" dimers of inducible nitric oxide synthase. Proc Natl Acad Sci U S A. 2003; 100: 14263–14268. http://dx.doi.org/10.1073/pnas.2435290100

[9] Tham SJ, Chang CD, Huang HJ, Lee YF, Huang TS, Chang CC. Biochemical

characterization of an acid phosphatase from Thermus thermophilus. Biosci. Biotechnol. Biochem 2010; 74: 727–735. http://dx.doi.org/10.1271/bbb.90773

[10] Pinato O, Musetti C, Farrell NP, Sissi C. Platinum-based drugs and proteins: reactivity and relevance to DNA adduct formation. J. Inorg. Biochem. 2013; 122: 27–37. http://dx.doi.org/10.1016/j.jinorgbio.2013.01.007

[11] Nowakowski AB, Wobig WJ, Petering DH. Native SDS-PAGE: high resolution electrophoretic separation of proteins with retention of native properties including bound metal ions. Metallomics. 2014;6: 1068–1078. http://dx.doi.org/10.1039/C4MT00033A

References in Supplementary Table B

[12] Solis C, Oliver A, Andrade E. PIXE analysis of proteins from a photochemical center. Nucl. Instr. Meth. Phys. Res. Sect. B, 1998; 136: 928–931. Available: http://www.sciencedirect.com/science/article/pii/S0168583X97008963

[13] Li J, Lee YJ, Assmann SM. Guard Cells Possess a Calcium-Dependent Protein Kinase That Phosphorylates the KAT1 Potassium Channel 1. Plant Physiol. 1998; 116: 785–795. http://dx.doi.org/10.1104/pp.116.2.785

[14] Yoon GM, Cho HS, Ha HJ, Liu JR, Lee HP. Characterization of NtCDPK1, a calcium-dependent protein kinase gene in Nicotiana tabacum, and the activity of its encoded protein. Plant Mol Biol. 1999; 39: 991–1001.http://dx.doi.org/10.1023/A:1006170512542

[15] Köhler C, Neuhaus G. Characterisation of calmodulin binding to cyclic nucleotide-gated ion channels from Arabidopsis thaliana. FEBS Lett. 2000; 471: 133–136. doi:10.1016/S0014-5793(00)01383-1

[16] Allardyce CS, Dyson PJ, Abou-Shakra FR, Birtwistle H, Coffey J. Inductively coupled plasma mass spectrometry to identify protein drug targets from whole cell systems. Chem. Commun 2001; 24:2708–2709. http://dx.doi.org/10.1039/b108418f

[17] Gao Y, Chen C, Zhang P, Chai Z, He W, Huang Y. Detection of metalloproteins in human liver cytosol by synchrotron radiation X-ray fluorescence after sodium dodecyl sulphate polyacrylamide gel electrophoresis. Anal Chim Acta. 2003; 485: 131–137. doi:10.1016/S0003-2670(03)00347-7

[18] Binet MRB, Ma R, McLeod CW, Poole RK. Detection and characterization of zinc- and cadmium-binding proteins in Escherichia coli by gel electrophoresis and laser ablation-inductively coupled plasma-mass spectrometry. Anal Biochem. 2003; 318: 30–38. doi:10.1016/S0003-2697(03)00190-8

[19] Weseloh G, Kuhbacher M, Bertelsmann H, Ozaslan M, Kyriakopoulos A, Knochel A, et al. Analysis of metal-containing proteins by gel electrophoresis and synchrotron radiation X-ray fluorescence. J Radioanal Nucl Chem. 2004; 259: 473–477. doi:10.1023/B:JRNC.0000020921.66046.1c

[20] Becker JS, Zoriy M, Becker JS, Pickhardt C, Damoc E, Juhacz G, Palkovits M, Przybylski M. Determination of phosphorus-, copper-, and zinc-containing human brain proteins by LA-ICPMS and MALDI-FTICR-MS. Anal. Chem. 2005; 77: 5851–5860. http://dx.doi.org/10.1021/ac0506579

[21] Verbi FM, Arruda SCC, Rodriguez APM, Perez CA, Arruda MAZ. Metal-binding proteins scanning and determination by combining gel electrophoresis, synchrotron radiation X-ray fluorescence and atomic spectrometry. J Biochem Biophys Methods. 2005; 62: 97–109. doi:10.1016/j.jbbm.2004.09.008

[22] Krause-Buchholz U, Becker JS, Zoriy M, Pickhardt C, Przybylski M, Rödel G, Becker JS. Detection of phosphorylated subunits by combined LA–ICP–MS and MALDI–FTICR–MS analysis in yeast mitochondrial membrane complexes separated by blue native/SDS-PAGE. Int. J. Mass Spectrom. 2006; 248: 56–60 http://dx.doi.org/10.1016/j.ijms.2005.10.006

[23] Finney L, Chishti Y, Khare T, Giometti C, Levina A, Lay PA, et al. Imaging metals in proteins by combining electrophoresis with rapid X-ray fluorescence mapping. ACS Chem Biol. 2010; 5: 577–587. doi:10.1021/cb1000263

[24] Jiménez MS, Rodriguez L, Gomez MT, Castillo JR. Metal-protein binding losses in proteomic studies by PAGE-LA-ICP-MS, Talanta 2010; 8: 241–247. http://dx.doi.org/10.1016/j.talanta.2009.11.064

[25] Romeis T, Franz S, Ehlert B, Liese A, Kurth J, Cazale A. Calcium-Dependent Protein Kinase CPK21 Functions in Abiotic Stress Response in Arabidopsis thaliana. 2011; 4. doi:10.1093/mp/ssq064

[26] Mena ML, Moreno-Gordaliza E, Moraleja I, Canas B, Gomez-Gomez MM. OFFGEL isoelectric focusing and polyacrylamide gel electrophoresis separation of platinum-binding proteins. J Chromatogr A. 2011; 1218: 1281–1290. doi:10.1016/j.chroma.2010.12.115

[27] Mena ML, Moreno-Gordaliza E, Gómez-Gómez MM. TCEP-based rSDS-PAGE and nLC-ESI-LTQ-MS/MS for oxaliplatin metalloproteomic analysis, Talanta 2013;116: 581–592. http://dx.doi.org/10.1016/j.talanta.2013.06.044

[28] Pinato O, Musetti C, Farrell NP, Sissi C. Platinum-based drugs and proteins: reactivity

and relevance to DNA adduct formation. J. Inorg. Biochem. 2013; 122: 27–37. http://dx.doi.org/10.1016/j.jinorgbio.2013.01.007

[29] Nowakowski AB, Wobig WJ, Petering DH. Native SDS-PAGE: high resolution electrophoretic separation of proteins with retention of native properties including bound metal ions. Metallomics. 2014;6: 1068–78. doi:10.1039/c4mt00033a

[30] Moscatiello R, Zaccarin M, Ercolin F, Damiani E, Squartini A, Roveri A, Navazio L. Identification of ferredoxin II as a major calcium binding protein in the nitrogen-fixing symbiotic bacterium *Mesorhizobium loti*, BMC Microbiol. 2015; 15:1–9. http://dx.doi.org/10.1186/s12866-015-0352-5

References for Table C

[31] Hutchens TW, Nelson RW, Yip T. Evaluation of Peptide / Metal Ion Interactions by UV Laser Desorption Time-of-flight Mass Spectrometry. J. Mol. Recogn. 1991; 4: 151–153. doi: 10.1002/jmr.300040407

[32] Nelson RW, Hutchens TW. Mass spectrometric analysis of a transition-metal-binding peptide using mass spectrometry. A demonstration of probe tip chemistry. Rapid Commun Mass Spectrom 1992; 6: 4–8. doi: 10.1002/rcm.1290060103

[33] Woods AS, Buchsbaum JC, Worrall TA, Berg JM, Cotter RJ. Matrix-Assisted Laser Desorption/Ionization of Noncovalently Bound Compounds. Anal Chem. 1995; 67: 4462–4465. doi:10.1021/ac00120a005

[34] Hornshaw MP, McDermott JR, Candy JM. Copper binding to the N-terminal tandem repeat regions of mammalian and avian prion protein. Biochem Biophys Res Commun. 1995; 207: 621–629. doi:10.1006/bbrc.1995.1233

[35] Kaltashov IA, Cotter RJ, Harry W, Ketner GW. Ferrichrome: Surprising stability of a cyclic peptide-FeIII complex revealed by mass spectrometry. J Am Soc Mass Spectrom 1997; 8: 1070–1077. doi:10.1016/S1044-0305(97)00128-1

[36] Masselon C, Salih B, Zenobi R. Matrix-assisted laser desorption/ionization Fourier transform mass spectrometry of luteinizing hormone releasing hormone-metal ion complexes. J Am Soc Mass Spectrom. 1999; 10: 19–26. doi:10.1016/S1044-0305(98)00128-7

[37] Lehmann R, Zenobi R, Vetter S. Matrix-assisted laser desorption/ionization mass spectra reflect solution-phase zinc finger peptide complexation. J Am Soc Mass Spectrom. 1999; 10: 27–34. doi:10.1016/S1044-0305(98)00116-0

[38] Cerda BA, Cornett L, Wesdemiotis C. Probing the interaction of alkali and transition metal ions with bradykinin and its des-arginine derivatives via matrix-assisted laser desorption/ionization and postsource decay mass spectrometry. Int J Mass Spectrom. 1999; 193: 205–226. doi: 10.1016/S1387-3806(99)00164-5

[39] Qin K, Yang Y, Mastrangelo P, Westaway D. Mapping Cu(II) Binding Sites in Prion Proteins by Diethyl Pyrocarbonate Modification and Matrix-assisted Laser Desorption Ionization-Time of Flight (MALDI-TOF) Mass Spectrometric Footprinting. J Biol Chem. 2002; 277: 1981–1990. doi:10.1074/jbc.M108744200

[40] Becker JS, Zoriy M, Becker JS, Pickhardt C, Damoc E, Juhacz G, et al. Determination of phosphorus-, copper-, and zinc-containing human brain proteins by LA-ICPMS and MALDI-FTICR-MS. Anal Chem. 2005; 77: 5851–5860. doi:10.1021/ac0506579

[41] Becker JS, Zoriy M, Przybylski M, Becker JS. Study of formation of Cu- and Zn-containing tau protein using isotopically-enriched tracers by LA-ICP-MS and MALDI-FTICR-MS. J Anal At Spectrom. 2007; 22: 63–68. doi:10.1039/B609419H

[42] Becker JS, Zoriy M, Przybylski M, Becker JS. High resolution mass spectrometric brain proteomics by MALDI-FTICR-MS combined with determination of P, S, Cu, Zn and Fe by LA-ICP-MS. Int J Mass Spectrom. 2007; 261: 68–73. doi:10.1016/j.ijms.2006.07.016

[43] Hortal AR, Hurtado P, Martínez-Haya B. Matrix-assisted laser desorption mass spectrometry of gas-phase peptide–metal complexes. Appl Phys A. 2008; 93: 935–939. doi:10.1007/s00339-008-4739-0

[44] Becker JS, Mounicou S, Zoriy M V., Becker JS, Lobinski R. Analysis of metal-binding proteins separated by non-denaturating gel electrophoresis using matrix-assisted laser desorption/ionization mass spectrometry (MALDI-MS) and laser ablation inductively coupled plasma mass spectrometry (LA-ICP-MS). Talanta. 2008; 76: 1183–1188. doi:10.1016/j.talanta.2008.05.023

[45] Karmakar S, Das KP. Identification of histidine residues involved in Zn2+ binding to αA- and αB-Crystallin by chemical modification and MALDI TOF mass spectrometry. Protein J. 2012; 31: 623–640. doi:10.1007/s10930-012-9439-0

[46] Jayasinghe SB, Caruso JA. Preliminary investigation of Cu-containing proteins in seeds of Brazil nuts by ICPMS and MALDI-MS methods. Int J Mass Spectrom. 2013; 356: 33–36. doi:10.1016/j.ijms.2013.09.014
